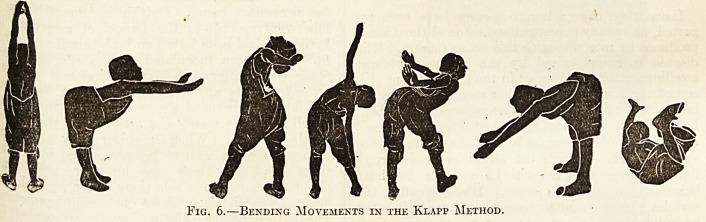# The Klapp Treatment of Scoliosis

**Published:** 1911-01-14

**Authors:** 


### SPECIAL ARTICLES.

### THE KLAPP TREATMENT OF SCOLIOSIS.

The "crawl" treatment of scoliosis which is associated with the name of Professor R. Klapp is now generally recognised as a most valuable aid to the orthopædist, and is being used at all up-to-date institutions in England, in America, and on the Continent. Nevertheless, though its merits have been demonstrated to the profession for the last six years, it is a method which is as yet comparatively unknown to the general practitioner in this country. This is unfortunate, for the treatment is in many ways eminently suitable for better-class patients in private practice who can devote the necessary time and attention to it which the details demand, and even in the case of poor and club patients the treatment can be carried out efficiently without unnecessary expense.

### At the Ziegel Street Clinic.

The home of the Klapp treatment, formerly at the Surgical Clinic, at Bonn, was transferred, a few years ago, to the larger and better-equipped orthopædic department of the Royal Surgical Clinic in the Ziegelstrasse at Berlin. Here Professor Klapp, assisted by his right-hand orthopædist, Dr. Fränkel, treats several score of children with all degrees of

Fig. 1.

Fig. 2.

lateral curvature every day, and the crawl treatment can be witnessed with all its latest and best modifications. To anyone accustomed to the often haphazard and happy-go-lucky methods which prevail in English out-patient departments so far as orthopædic cases are concerned, nothing can be more enlightening than the routine that is here followed. Every new case is most carefully and methodically examined. Each patient is completely stripped and accurate measurements are taken and preserved in a record, which, with the photograph of the case during various stages of the treatment, is always available for reference. And here it may be pointed out that only by following such a methodical course of inspection can the practitioner do justice to the case or know whether the treatment is benefiting his patient or not. There is a prejudice against completely stripping patients who have obvious lateral curvature: it is regarded as "an unnecessary indignity which is not justified when the condition is obvious," according to one English authority. This is ridiculous nonsense. No considerations of false conventionality should interfere with the rigorous examination of a case of lateral curvature, and it is only by completely stripping the patient that the practitioner can obtain a correct appreciation of the severity of the case before him. We have laid stress on this point, usually regarded as a small one, because we are fully aware that such routine examination is not general in London hospitals, and students are trained to take a somewhat careless estimate of cases of general curvature. To give only one instance out of many, there are patients now under treatment at some of our hospitals whose lateral curvatures are dependent on uncorrected and overlooked deformities of the foot: such mistakes would never have arisen with proper examination, but is relatively common when

Fig. 3.

the patient strips merely the back and shoulders and the contour of the lower part of the body is hidden by a wealth of fallen clothes.

### Some Points to be Remembered.

It is unnecessary here to go into the symptomatology, pathology, and general course of scoliosis. It may, however, be pointed out that there is no essential difference between lateral curvature and scoliosis and the academic distinction drawn in most text-books is fallacious. In every case of lateral curvature which is exaggerated—for lateral curvature itself is wholly physiological, and it is only when the normal curves are grossly increased or abnormal ones are added that the case should be treated—there is rotation, even though it may not be obvious on ordinary examination. As a preliminary to treatment every case should be thoroughly examined, and notes of the examination should be kept for record purposes. In this examination special attention should be paid to the legs and feet, the brim of the pelvis, the ribs and clavicles, and the neck. There are a few obvious conditions which should never be left untreated, as without their correction no treatment of the scoliosis

Fig. 4.

will meet with the slightest success. These are wry neck and the various forms of flat and club foot; ulcers or sinuses in the thorax or neck, and pelvic deformities or irritation in female patients. Such cases are all unsuitable for the crawl treatment until the exciting cause of the scoliosis has been removed. In most cases the accessory treatment is fairly easy: for the wry neck subcutaneous sterno-mastoid myotomy should be done, the foot deformities should be corrected and the sinuses healed before the treatment with exercises is begun.

### The Crawl Treatment.

Having satisfied himself that the case is one of "pure" scoliosis" primarily dependent upon muscular weakness, the practitioner may try the crawl treatment with every confidence in ultimate success, provided that the exercises are systematically and regularly done. At first these exercises should be carried out under the personal supervision of the medical attendant; later on an intelligent mother, father, or elder brother or sister, the district nurse, or an out-patient nurse where the patient is attending a hospital, may be trusted to see that they are properly practised. In every case, however, where the practitioner delegates this duty to another he should assure himself that the details of the exercises are thoroughly understood by whomsoever takes charge of the patient. At Professor Klapp's clinic this supervision is exercised by trained turnlehrerinnen or gymnastic nurses, and it is a

Fig. 5.—Dress Worn by School Child at Berlin Clinic.

pleasure to witness the expert manner in which they handle their several squads. In no case, again, should the child be permitted to do the exercises by itself without supervision, no matter how expert in crawling it has become, because it is impossible for the patient to know whether the poise of head and shoulders is over- or under-done. That can only be correctly judged by a trained bystander, who can correct any faults in position as soon as they are observed.

### The Essentials of the Crawl.

The essentials of the Klapp crawl method consist in a series of lateral bending movements which have for their object the mobilisation of certain segments of the spinal column. Ordinarily the spinal column of the child with lateral curvature is comparatively rigid. The rigidity indeed is not absolute: it cannot for a moment be compared with that which results from organic disease of the vertebræ. But it is a "stiff spine" lacking the suppleness that is to be noticed in the vertebral column of normal children with a well-developed musculature. The crawl treatment attempts to strengthen the musculature of the back, while at the same time it gets rid of this stiffness which affects the patient's posture and carriage. The exercises devised towards that end are of two kinds, crawling and bending movements being the main characteristics of each set of exercises.

### A Lesson from Natural History.

In the first set, the child learns to crawl in much the same way as a dog or cat runs, and not quite in the same way as a baby crawls. The accompanying illustrations, modified from Professor Klapp's text-book on the subject * will explain these movements almost better than mere description can. In the first movement the one arm is stretched out, the child being on hands and knees on a hard, polished floor, or, better still, on a smooth lawn or on a street pavement if nothing better is available. The leg on the same side is advanced while the leg on the opposite side is stretched out. At the same side the head is vigorously turned to the side of the convexity of the main lateral curve. Figs. 1 to 4 show this stage. By alternating these movements the patient progresses forward in much the same manner as a dog. The practitioner would do well to practise the crawl himself in the quiet seclusion of his own bedroom: ten minutes' practice will convince him of the fact that the exercise is fatiguing and eminently calculated to mobilise not only his spinal, but all his joints!

### A Note on Clothing.

A word must be said about the child's dress. Fig. 5 shows the dress worn by female patients at the Berlin clinic. Usually the skirt is discarded, and the exercises are done in undershirt and drawers; in the case of boys in singlet and knickers, or "shorts." On the hands and knees are worn stiff leather pads to prevent abrasions of the skin in these situations. Such pads can be easily manufactured in ordinary private practice out of a pair of thick, old stockings, covered with cardboard, leather, or felt, and strapped on over the hands and knees. The essentials are that the clothing should be loose and accommodate itself to the body movements without in any way hindering or interfering with them.

### Group Exercises.

The second group of exercises are such as are represented by Fig. 6. They are body movements analogous to Swedish gymnastics, and are intended to exaggerate the curves induced by the first group. They consist principally of bending (anterior, posterior and lateral) movements by the patients themselves, or of similar, but more forcible, movements where the patients are helped by the instructor or by being strapped down to an exercise board. The antero-posterior and l[illegible]eral movements must be done in succession, and i[illegible]ery case great

* *Die functionelle Behandlung d*[illegible]*ose.* Gustav Fischer. Jena 1910.

attention must be paid to the poise of the head and shoulders.

Variations of these exercises easily suggest themselves. Thus one of the finest for children with lateral curvature is swimming, and they should be encouraged to devote a part of their time every day in summer at least to this admirable recreation. The old-fashioned ring swinging and bar exercises may also be included in the treatment, as although by themselves they do not do much towards improving the scoliosis, they are valuable auxiliaries to the crawl treatment.

THE IMPORTANCE OF SYSTEMATIC EXERCISE.

It has already been stated that all these exercises must be regular and systematic. In cases of slight scoliosis one crawl of half an hour's duration, with frequent resting intervals, in which the child must lie flat on its stomach or back, is sufficient: in more severe degrees of curvature there should be several crawls during the day, with exercises of the second group on the bench or with the instructor fixing one part of the child's body and with auxiliary exercises. In a few cases suspension and fixation in a plaster bed at nights must be done, but apparatus is to be avoided wherever possible. The treatment must be carried on for months, even years in bad cases. In minor degrees of deformity a few weeks' systematic treatment usually produce the most excellent results in the way of improvement. A record, with photographs and measurements, should be kept, and the degrees of improvement noted. Children undergoing the treatment are best kept away from school, because much of the good done by the crawls is destroyed by the comparatively long period of fixation in an unnaturally stiff position on the conventional benches which board school, and even private school, pupils have to undergo. At Berlin this abstention from school is insisted upon, and every child who comes to the clinic for treatment receives a certificate to the effect that he or she is unfit to go to school until further notice. A statement that the child is suffering from "severe scoliosis" is generally quite sufficient for the teacher.

FIG. 6.—BENDING MOVEMENTS IN THE KLAPP METHOD.

INDICATIONS FOR TREATMENT.

The Klapp treatment is applicable to adults as well as to children, but the prospects in an adult patient who has been scoliotic since childhood are poor, and the crawls and accessory treatment must be persevered in for a long time before any improvement is noticeable.

Patients between six and twelve years of age do the best: before the lower age they cannot easily be taught to do the rather complicated crawl movements, and after twelve the cases are usually severe. Nevertheless, we have seen excellent results with young adult patients; while with care and attention even a child of four may be taught to "crawl" in a proper way. The children usually take readily to the method: they like the crawl, especially when it is performed socially with a number of patients. Solitary crawls are to be avoided wherever possible.

The crawl treatment is one which can thoroughly be recommneded to the attention of the general practitioner, to whom it appears to be as little known as the hyperæmic method of Bier. The results obtained are far superior to those given by the older and more happy-go-lucky methods, and no expensive or cumbrous apparatus is needed. What is necessary is care, with individual attention, and unless the practitioner is prepared to give this to his scoliotic patients he had far better leave them alone.

## Figures and Tables

**Fig. 1. f1:**
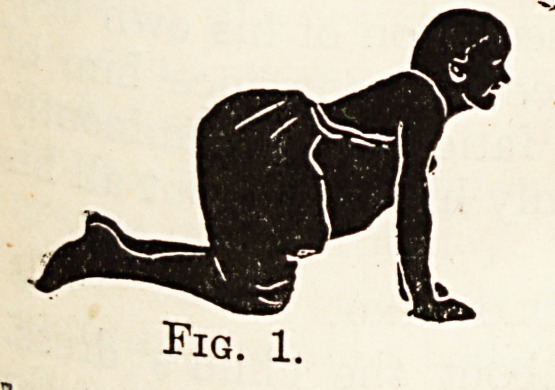


**Fig. 2. f2:**
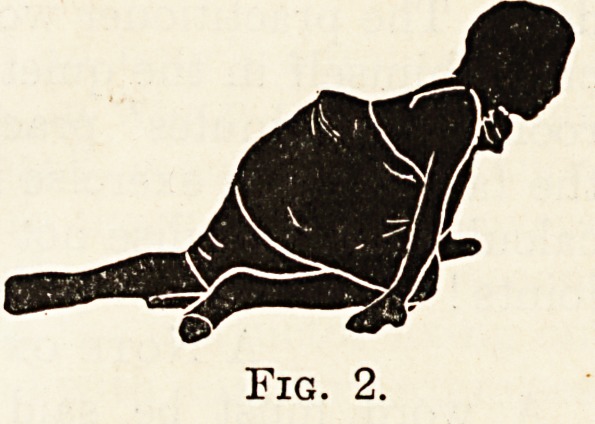


**Fig. 3. f3:**
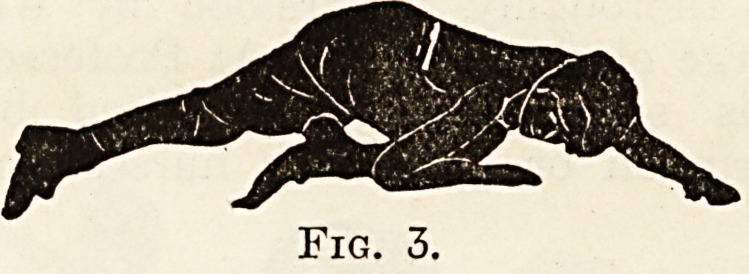


**Fig. 4. f4:**
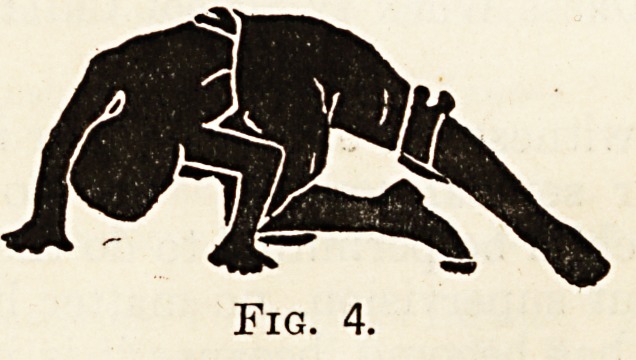


**Fig. 5. f5:**
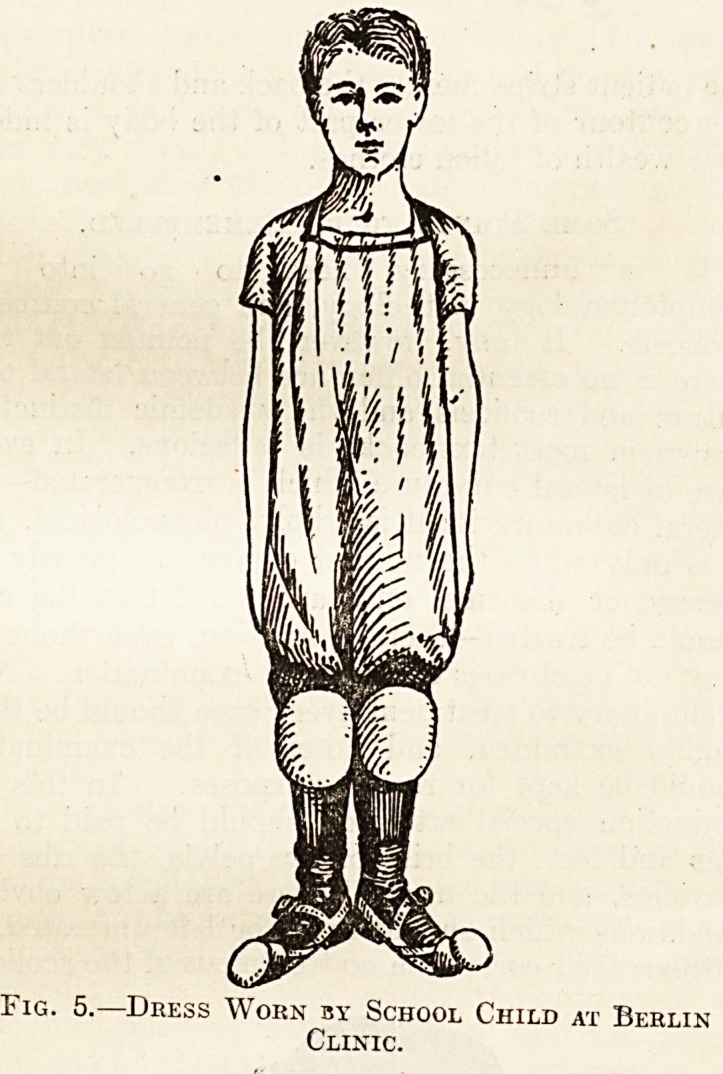


**Fig. 6. f6:**